# Recovery of ro-pax ferry traffic from covid-19 under tightening environmental regulations: case Helsinki-Tallinn

**DOI:** 10.1186/s41072-022-00112-x

**Published:** 2022-05-07

**Authors:** Ulla Tapaninen, Riina Palu

**Affiliations:** 1 Estonian Maritime Academy, Tallinn University of Technology, Kopli 101, 11712 Tallinn, Estonia; 2 Kouvola Unit, LUT University, Prikaatintie 9, 45100 Kouvola, Finland

**Keywords:** Covid-19, Decarbonisation, Ro-ro traffic, Ro-Pax traffic, Helsinki-Tallinn

## Abstract

In 2020, the number of passengers on international ro-pax ferries collapsed due to the pandemic caused by the Covid-19 virus and subsequent travel restrictions. At the same time, both the International Maritime Organisation and the European Union are setting stringent regulations on carbon dioxide emissions from ships. In this research, we look at what options companies offering ro-pax services have to recover from the Covid-19 pandemic under-tightening environmental regulations and the future options for the industry. The case under study is ro-pax ferry traffic between Helsinki and Tallinn. It is one of the busiest international passenger ferry connections in the world. The economics of transport are based on large high-speed vessels, the combination of passengers and cargo, and sales onboard. We created four scenarios for the traffic: to continue the same high-speed ro-pax system as in 2019, to reduce the number of vessels, to switch to new types of ships, to slow down the speed of the vessels or to divide traffic into faster and slower ships. The research contributes to discussion of competitive strengths of ro-pax transport.

## Introduction

In March 2020, the Covid-19 virus gave a brutal hit to the traffic in the world (March et al. 2021). The world retreated to quarantine, and many countries closed their borders, especially to tourism and passenger traffic. In maritime transport, cruise traffic and ro-pax ferries (roll on -roll off passenger vessels) were particularly affected by the pandemic. In 2020, cruise traffic volumes in European Union (EU) fell by 85 per cent from the 2019 level, and passenger traffic in the EU by 39 per cent (European Maritime Safety Agency, EMSA 2021).

At the same time, the International Maritime Organization (IMO) has continued to tighten its targets for reducing greenhouse gas (GHG) emissions. In 2018, the IMO decided to reduce the carbon intensity of international shipping, i.e. carbon dioxide (CO_2_) emissions from maritime transport performance (grams / tonne-kilometre), by at least 40% between 2008 and 2030.

In July 2021, the European Commission adopted a series of legislative proposals setting out how it intends to achieve climate neutrality in the EU by 2050, including the intermediate target of at least 55% net reduction in greenhouse gas emissions by 2030. The package proposes to revise several pieces of EU climate legislation, including the EU Emissions Trading System (ETS), Effort Sharing Regulation, transport and land use legislation, and sets out in real terms how the Commission intends to reach EU climate targets under the European Green Deal. Maritime traffic is part of this package.

In this research, we look at the recovery of ro-pax ferry traffic from the constraints caused by the Covid-19 pandemic and consider future developments. Tightening international environmental regulations pose a unique challenge for the traffic. We are studying the situation of one of the world’s busiest international passenger traffic routes, i.e. Helsinki-Tallinn traffic. The target year of the study is 2030, when the effects of pandemic will be overcome, but the environmental regulations will start to have a significant influence on the business.

The research questions are:

1. How has the Covid-19 pandemic affected ro-pax ferry traffic, which is mainly dependent on passengers?
2. How will future environmental regulations affect ro-pax ferry traffic?
3. What are the options for ro-pax ferry services to recover from both the tightening environmental regulations and the Covid-19 pandemic and what are the options for the future of this service?

The methodology in this study was first to analyse separately the factors affecting the Helsinki-Tallinn traffic, figure out the most significant ones under change and clarify their possible effects on this traffic. Finally the combined effect of the two major influential factors were analysed. The most significant factors describing future developments are the volume of passenger traffic and the impact of environmental regulations. The combined effect of these are studied in four different scenarios, each of them assessed separately.

The second section of this research shows literature review of short sea shipping, and namely previous research Helsinki-Tallinn traffic. Third section looks at the tightening of environmental regulations and their effects on ro-pax ferry traffic. In the fourth section, we look at how Helsinki-Tallinn traffic has developed during the last 30 years and which factors affect the growth of the traffic. We also look at how the Covid-19 pandemic has affected the traffic. In the fifth section, we consider different future options for Helsinki-Tallinn traffic and expand the perspective to ro-pax ferry traffic in general. In the sixth section, the conclusion of the research are given. Finally, in the seventh section, we discuss how this research contributes the the previous research and about the limitations of the research as well as further research opportunities.

## Success factors of short sea shipping

European union has been trying to increase the share of short-sea shipping (see e.g. EC 2015), but the share of short-sea shipping has remained stable during years (van der Boss et al. 2018). Musso et al. (2010) in their thorough presentation of short-sea shipping state that the profound problem in European short-sea shipping is that it has to compete with road transport on cost, speed, flexibility, reliability, attractive sailing times, maintained transit times, and guarantee of delivery. This is also stated by Russo et al. (2016) and Suarez-Aleman et al*.* (2015).

**Fig. 1:**
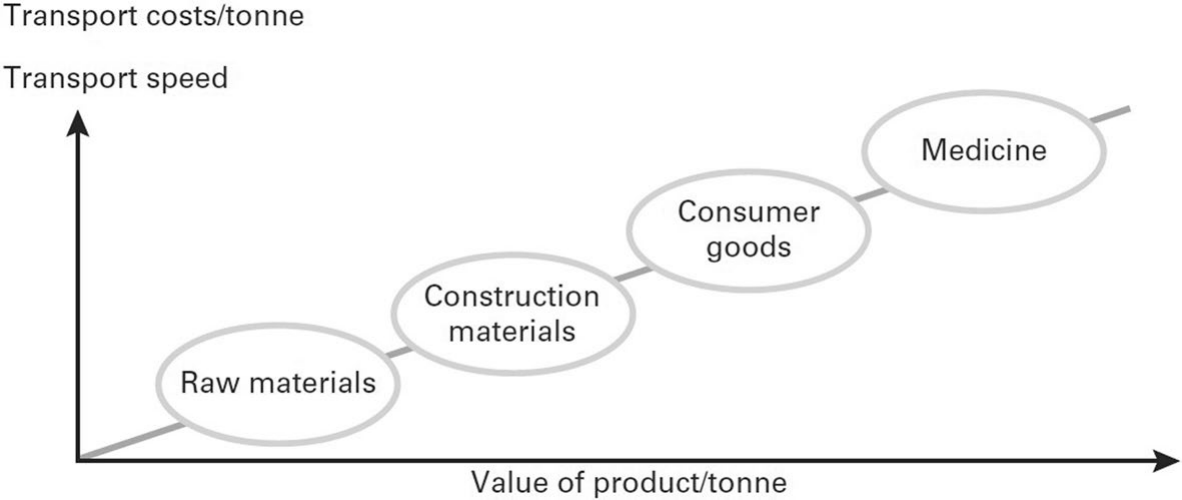
The relationship between product value and transport speed and costs. *Source*: Tapaninen (2020)

Alford and Bangs (1944) created the concept of annual cargo holding costs. Based on this study, Yang et al. (2015) the estimated annual holding cost of general cargo between 23 and 26% of invoice value and range of 42–45% for high-tech cargo. The mode of transport of a product is most often determined by the time spent on transport: the more expensive and lighter the product, the faster it is wanted to be transported and the more there is will to pay for the cost, see Fig. 1 (Tapaninen 2020).

In other words, the more expensive the product, the greater the requirement for the speed of the trip. In their recent study. Zheng et al. (2021) show that even though ro-ro (roll-on-roll-off) traffic is considerably more expensive than other modes of sea transport, e.g. lo-lo (lift on–lift off) and bulk transport, ro-ro vessels has a substantial impact on the reduction in total logistics cost of the entire supply chain cycle.

Compared with lo-lo operations, ro-ro vessels can be loaded and unloaded much faster, and therefore it reduces the port time. This is important for the vessel’s turnaround, but even more, the ro-ro transports are suitable for cargo that need of fast transit time. The ro-ro transport allows quick modal shift from sea transportation to road transportation. However, the reduced transit time of ro-ro transport increases the freight rate, as ro-ro vessels have limited loading capacity compared to lo-lo vessels same size.

## Decarbonising shipping

The shipping industry (international, domestic and fishing) is responsible for 1076 million tonnes in 2018 of GHG emissions — including CO_2_, methane (CH_4_) and nitrous oxide (N_2_O), expressed in CO_2_e. As a result, the share of shipping emissions in global anthropogenic emissions has increased from 2.76% in 2012 to 2.89% in 2018 (IMO 2020). Even though the figure is not very big, the share will continue increasing if other energy production or transport sectors decrease rapidly their emissions.

In 2018, the IMO (International Maritime Organization) agreed upon reducing the carbon intensity of international shipping (to reduce CO_2_ emissions per transport work, as an average across international shipping, by at least 40% by 2030, pursuing efforts towards 70% by 2050, compared to 2008). As a result, the total annual GHG emissions from international shipping should be reduced by at least 50% by 2050 compared to 2008 (IMO 2021).

The IMO Marine Environment Protection Committee (MECP) meeting in June 2021 adopted short-term measures to reduce the carbon intensity of all ships by 40% by 2030, compared to 2008. With 2019 as the base year for the reference lines, the carbon intensity will decrease 11% by 2026. The rates for 2027–2030 will be decided as part of the review to be concluded by 1 January 2026.

In addition to IMO regulation, it has also been stated that stricter regulations are needed. In particular, the effectiveness of market-based measures has been studied carefully, as presented in Lagorvaudou et al. (2020), Wan et al. (2018) and Psaraftis et al*.* (2021). Dominioni (2018) states that if carefully designed, a cargo-based measure that covers the emissions released throughout the voyage to the cargo destination presents various advantages compared with other carbon pricing schemes.

In July 2021, the EU Commission proposed to include shipping emissions for the first time in the EU ETS (Emission Trading System). In addition, The Alternative Fuels Infrastructure Regulation requires that ships have access to clean electricity supply in major ports. The FuelEU Maritime Initiative will stimulate the uptake of sustainable maritime fuels and zero-emission technologies by setting a maximum limit on the greenhouse gas content of energy used by ships calling at European ports. The aim is to decrease GHG emission by 2% by 2025, 6% by 2030, 26% by 2040 and 75% by 2050. Details and consequences of this package are still under study and discussion (EU 2021).

These environmental regulations put shipping into a new situation. Stopford (2020) states that “*In the next 20 years the maritime industry must rebuild its cargo fleet. If this is done with the radical technologies now available, it will lead to the biggest change in ship design since steam replaced sail in the nineteenth century*.”

Presently, there is no ‘silver bullet’ to achieve the environmental targets in shipping, but multiple means are needed to reduce emissions of vessels. Means to reduce exhaust emissions from ships can be grouped into three groups, namely (i) fuel solutions, (ii) ship design and technological development, and (iii) ship type selection and ship speed choices.

There are numerous literature reviews of various ways to reduce carbon emissions of shipping, see, e.g. Bouman et al. (2017), Serra and Franello (2020) and Miola et al. (2011).

Balcombe et al. (2018) also give an overview of various means to decarbonise shipping. They state that ‘*liquefied natural gas (LNG) is reaching mainstream and provides 20–30% CO*_*2*_* reductions whilst minimising SO*_*2*_* and other emissions. Costs are favourable, but GHG benefits are reduced by methane slip, which varies across engine types. Biofuels, hydrogen, nuclear and carbon capture and storage (CCS) could all decarbonise much further, but each faces significant barriers around their economics, resource potentials and public acceptability. Regarding efficiency measures, considerable fuel and GHG savings could be attained by slow-steaming, ship design changes and utilising renewable resources. There is clearly no single route and a multifaceted response is required for deep decarbonisation.’*

The service life of a vessel is practically always more than 20 years, up to 30. Therefore, the ship designs already on the table must take the best possible environmental standards into account. Energy solutions are as emission-free as possible, and the ship’s energy consumption is kept to a minimum concerning the amount of cargo carried.

In particular, ro-ro and ro-pax ships consume the most energy per transport unit (tonne-NM) in the maritime sectors (see Table 1). Therefore, in principle, the economic burden of emissions reduction would be more significant for ro-ro and ro-pax ships than for other ship types. However, IMO has stated that the new regulations treat different types of ships differently. Rutherford et al. (2020) estimate that the IMO emission reductions required for the largest container vessels are up to 50%, while only about 5% reductions are required for smaller ro-ro vessels.

**Table 1:** Emissions reported in MRV for different shipping segments

Ship type	Emissions	Emission per distance	Emissions per transport work
	Mtonnes CO_2_	kg CO_2_/NM	g CO_2_ / tonne-NM
Bulk	18.1	290	8.48
Container	44.4	570	20.13
General cargo	6.13	185	28.02
Oil Tanker	18.1	435	8.82
Ro-ro	6.06	338	91.03

*Source*: Mellin et al. 2020

In the Baltic Sea, new fuel solutions are currently the fastest-growing operation. Baltic ro-pax shipping companies have been pioneers in the use of low-emission LNG fuel, see, e.g. Turku (2017), Viking Line (2018) and Wasaline (2021). In addition, emissions have been reduced by connecting the ship to shore-side electricity during port calls. Ports have built onshore electricity capacity and are preparing to distribute new marine fuels, see, e.g. Invest in Estonia (2021).

At the moment, it seems that the small urban waterway ferries are being replaced by electric ones, e.g. VisitCopenhagen (2021), Helsinki (2021) and Maritime executive (2017). Lately, there has also been much discussion of hydrogen, see, e.g. Invest in Estonia (2021). Large ships are switching to batteries with natural gas, see, e.g. Finnlines (2020), and later either hydrogen or ammonia, see, e.g. FuelCellWorks (2021). Many marine machine manufacturers are developing engines that can utilise various fuel types, e.g. Wärtsilä (2021). Emissions can also be reduced through operational measures, the most effective of which is to reduce the ship’s cruising speed, see e.g. Cariou (2011), Tezdogan et al. (2015) and Dgiulia et al*.* (2021).

## Traffic between Helsinki and Tallinn

Due to the geographic position Finland is very dependent on sea traffic and competition with road traffic is minimal, except for Russian trade and transit traffic.

In 2020 83 per cent of Finnish foreign trade was carried out by sea (Statistics Finland 2021) in comparison to only 55 per cent of the Estonian foreign trade through ports (Statistics Estonia 2021a, 2021b). In principle, in Finland only the transports to and from Russia are carried out by road or rail. The shortest sea export and import routes of Finland are to Sweden and Estonia, while Helsinki-Tallinn route makes 63 per cent market share of sea-based truck traffic between Finland and other countries (Statistics Finland 2021).

The academic studies about Helsinki-Tallinn traffic is limited. Sundberg et al. (2011) studied the Helsinki-Tallinn route. They found that during the period 2002–2010 the volume of the seaborne cargo traffic between Finland and Estonia had increased significantly while the trend of the trade volume between Finland and Estonia has remained nearly constant. This indicates that the route via Estonia is increasingly used in the Finnish foreign trade. Similar results are presented by Hilmola (2014). Because the ports of Helsinki and Tallinn are the main ports in the cargo traffic between Finland and Estonia, the role of the Helsinki-Tallinn route as a sea leg in the hinterland connections of Finland has increased. The growth of the cargo volume on the Helsinki-Tallinn route was estimated to continue on the annual level of 10% during the next couple of years.

The study made by Sundberg et al. (2011) also shows that the fast and reliable connections year-round on the Helsinki-Tallinn route have made it possible for service and logistics companies to reconsider their logistics strategies in a new way in the both sides of the Gulf of Finland. They claimed that the ro-pax concept is seen by the operators as the only economical profitable solution on the Helsinki-Tallinn route because cargo and passenger traffic are supporting each other.

Hilmola et al. (2015) have questioned the study of Sundberg et al. (2011) and studied Helsinki-Tallinn short sea route. They state that these trucking-based transportation chains have very poor performance in terms of CO_2_ emissions and fuel efficiency. Due to coming environmental regulations they suggest that the truck and semi-trailer-based transportation will be challenged by containers.

However in 2021 the increase in containers is not seen (Traficom 2022), but truck traffic continues in fast ro-pax -vessels. The fast ro-pax -vessels on the route rely on extremely fast turn-around times in ports (less than one hour) that would not be possible, if the cargo would be transported in containers.

**Fig. 2:**
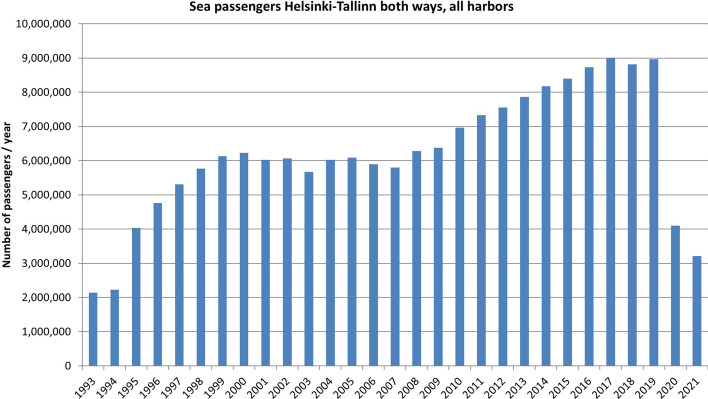
Sea passenger traffic between Helsinki and Tallinn (both ways). *Source*: Traficom (2011–2022) and its predecessors—author’s own compilation

Today, the Helsinki-Tallinn is one of the busiest maritime connections in the world. There has been maritime traffic between Finland and Estonia throughout the years; regular shipping was only suspended in 1940 and resumed in 1965. M/S Georg Ots, which was ordered for this service, began operation in this route in 1980 and ran until 2000. Estonia became independent in 1991, after which traffic between Finland and Estonia began to grow gradually up to 9 million one-way passenger trips per year in 2017, see Fig. 2.

Traffic grew with the same slow ro-pax ship concept until 2007 when Tallink brought its first fast ro-pax ferry into service. The first M/S Star travelled at a cruising speed of 24–27 knots, and the voyage time was shortened from 3.5 h to two hours. Before this, speed had been experienced in passenger traffic in the summer with fast catamaran vessels, but Tallink’s bold investment in new large high-speed ro-pax vessels significantly increased the number of passengers.

In 2019, there was four fast ro-pax ferries in the business. Tallink’s M/S Star (built 2007) and LNG -powered M/S Megastar (built 2017), Viking Lines M/S Viking XPRS (built 2008), and Eckerö Line’s Finlandia (built 2001). In addition, Tallink had a slower cruise vessel, the M/S Silja Europa (built 1993). In 2022, the M/S Star will be replaced by a new building, LNG-powered M/S MyStar with a capacity of 3000 passengers. In 2019, during the busiest season, i.e. during the summer holidays, departures between Helsinki and Tallinn accounted for 17 per day, which means departure once per hour, except at night.

The fast two-hour ro-pax crosses offered both tourists and commuters an easy and spirited crossing and eventually made the cabins almost useless. The concept had overwhelming advantages: due to a couple of hours of crossing and an hour of turning time, the ship could make 4–6 voyages per day and thus cover expensive capital costs and very high fuel costs due to speed. In addition, there were decks for cars and cargo, which, especially during the quiet wintertime, generated part of the ship’s revenue. Other fast but expensive and weather-sensitive traffic concepts, such as helicopters from port areas and catamarans, gradually disappeared from Helsinki-Tallinn traffic.

**Fig. 3:**
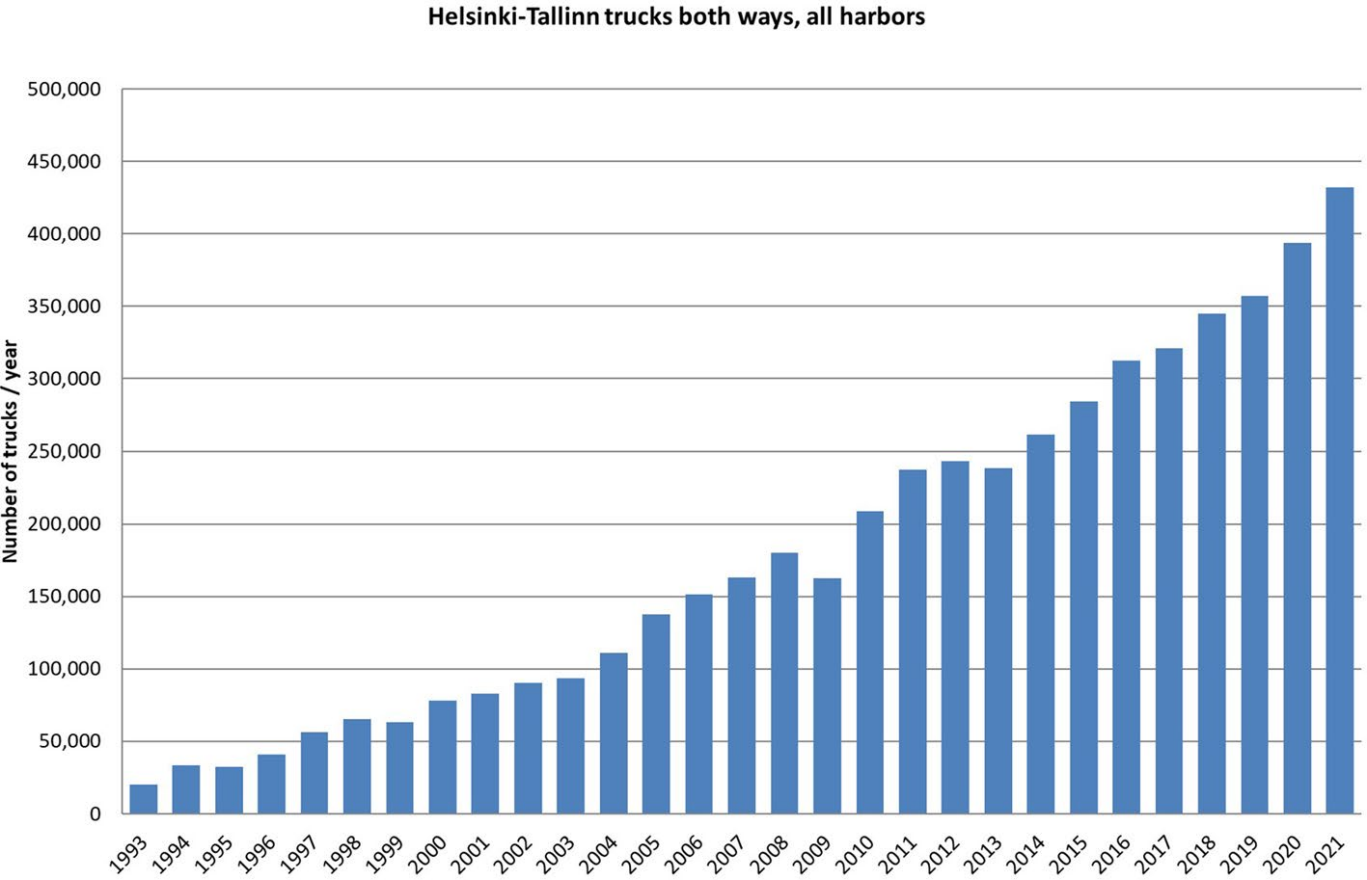
Truck traffic between Port of Helsinki and Port of Tallinn (units, all harbours, both ways). *Source*: Traficom (2011–2022) and its predecessors—author’s own compilation

Freight traffic and ro-pax transport also continued to grow steadily, and there was a need for more cargo capacity (see Fig. 3). As a result, Tallink started ro-ro traffic without passengers on M/S SeaWind in 2015 and Eckerö Line on M/S Finbo Cargo in 2019. These vessels operate from cargo harbours Helsinki’s Vuosaari and Tallinn’s Muuga in the outskirts of the cities, while the fast ro-pax ferries operate from city centres.

During this period, there have been several attempts to engage in the lorry, and trailer traffic between Hanko and Paldiski situated further from the capital areas, the most recent being carried out by several operators in 2011–2020, but it ceased in 2020.

**Fig. 4:**
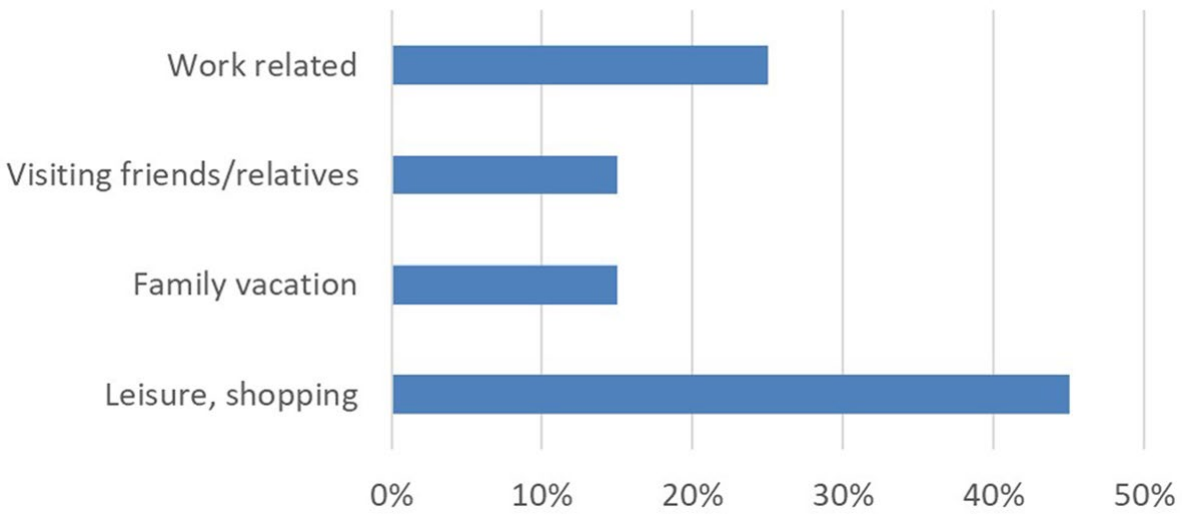
Reasons for travels for Helsinki-Tallinn trip 2019. *Source*: Ojala et al. (2020)—author’s own compilation

Figure 4 presents the main reasons for the passenger travels. 45% of the travellers between Estonia and Finland were on leisure or shopping trips. The share of business travellers was about 25% (Ojala et al. 2020). In 2018, in travels between Helsinki and Tallinn, the Finnish citizens made 58% of travellers, Estonian citizens 28% and other nationalities 14%. Almost 10% of Estonians were transit travel, which means that Estonians travelled through the port to the airport. (TAK 2019).

In 2020 Estonian maritime trade was 36 million tons (Statistics Estonia 2021a), and Finnish maritime trade 101 million tons (Statistics Finland 2021). There were 4.8 million tonnes transported between Helsinki and Tallinn (Port of Helsinki 2021). In a study made 2012 (Tapaninen 2012), it was found that about 2/3 of trucks were travelling only between Finland and Estonia and the rest 1/3 travelled further south to and from Finland. In Finland, about 42% of trucks remained in the Metropolitan area, and the rest travelled to other parts of the country.

The traffic was 95% lorries. The content was mainly consumer goods and industrial raw materials. The high frequency directed the busiest and most expensive products from Central Europe and Finland to this connection. A significant part of the goods going to the whole of Finland travelled on the line. The frequency was so good that consumer goods and industrial raw materials, which needed a fast connection to Europe, partially shifted to this line from direct shipping lines from Germany.

In addition, a significant share of goods was travelling only between two cities Helsinki and Tallinn, e.g. laundry or spare parts. In a sense, they were not export or import, but transport that can be characterised as intra-twin-city transports.

Three ro-pax companies are operating between ports of Helsinki and Tallinn and Helsinki and Stockholm passenger business. Tallink has the biggest market share, about 50% (Tallink 2021).

**Fig. 5:**
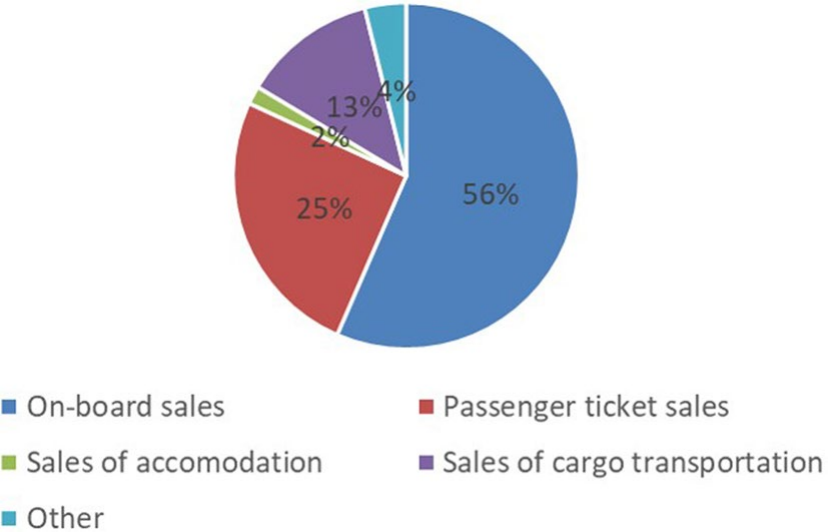
Tallink’s income from different geographical segments. *Source*: Tallink (2021)

**Fig. 6:**
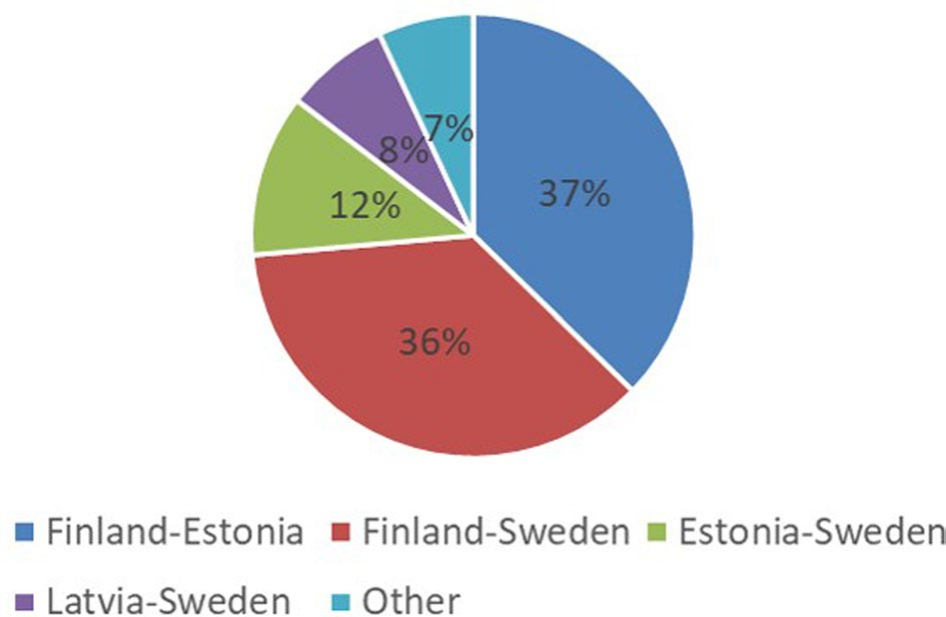
Tallink’s income from diffrent operating segments. *Source*: Tallink (2021)

The price of passenger tickets has remained very low, at best the round trip has been less than 10 euros remaining unchangingly low already from the beginning of 2000s. This has increased the demand for the line, especially as a leisure destination. However, the importance of cargo transport has been minor. For example, Fig. 5 shows that Tallink’s revenues in 2019 were 81% from passengers and 13% cargo decks (for all of its lines). The biggest income, over 56%, came from onboard sales (Tallink 2021). Figure 5 includes all Tallink’s Northern Baltic operations; Fig. 6 shows their division in various lines.

Traffic between Helsinki and Tallinn has been very profitable and growing. Even though there has been several attempts to have competing routes from Finnish ports Turku, Hanko and Kotka to Estonia, only the traffic between Helsinki and Tallinn has been able to stay in service the last 30 years. The profitability is due to high passenger-related income, but also the fact that they service combined two capital cities, and all their related cargo flows (to capital areas and via capital areas to other parts of the country, and in Estonian case, also transit Via Baltica), The profitability resulted also in constant investments of new fleet: an example of this is Tallink’s LNG-powered 2800-passenger M/S Megastar vessel started service in 2017 and a new M/S MyStar in 2022.

The Covid-19 pandemic did not treat passenger and cargo segments the same way. Truck and trailer traffic between Helsinki and Tallinn has grown almost steadily from 2005 to 2020. However, passenger numbers fell in 2019 by 54%, which had a huge impact on the financial performance of the companies.

The Finnish and Estonian states have supported ro-pax ferries between Helsinki and Tallinn with almost half of billion euros in 2020 and 2021 (Palu et al. 2021). Unfortunately, the result of the ferries has still been very poor. In 2020, Tallink Group’s result was EUR 108 million negative (net sales EUR 443 million) (Tallink 2021), Viking Line’s EUR 42 million negative (turnover EUR 189 million) (Viking Lin 2021) and Eckerö Line’s result for 2020 was minus EUR 34 million (turnover EUR 120 million) (Eckerö 2021).

## Reducing GHG-emissions in Helsinki-Tallinn traffic

Most of the Helsinki-Tallinn vessels are very fast, so the vessels consume much fuel compared to the amount of cargo. According to EMSA Thetis monitoring, reporting and verification of carbon dioxide emissions from maritime transport (MRV) report, in 2019, the emissions of the present ro-pax vessels vary from 485 to 733 kg CO_2_ per nautical mile (EMSA Thetis-MRV 2021).

In Decarbonising shipping” section, we went through how emissions from shipping can be reduced through ship design and technological development, fuel solutions, and changing the speed, size, or type of cargo of a ship. These measures also have their costs and implications more broadly, in some cases even to customer satisfaction.

It is tough to estimate how the shipping industry will adapt to the new GHG-emission regulations. Some indication can be obtained by looking at the Sulphur Directive, which came into force in 2015 in the Baltic Sea and was suspected of placing a heavy financial burden on shipping in the Baltic Sea (see Repka et al. 2017; Zis and Psaratis 2017). It is interesting to note that in Helsinki-Tallinn traffic, the volumes remained on a growing trajectory. This is mainly due to the increasing frequency of the traffic, being very attractive to truck traffic with high-value goods for consumers and industry.

The most economically viable options for reducing GHG emissions is in new buildings to reduce fuel consumption, such as designing the bottom of the ship, etc., that will simultaneously reduce bunker costs and emissions. Therefore, it is expected that the world maritime fleet will eventually produce lower emissions for economic reasons. On the other hand, the higher the charges come with emissions trading, the faster it will affect the fleet's renewal.

Low-emission fuels are still considerably expensive today compared to most fossil fuels, but as production increases and, for example, through emissions trading, the prices will fall (Solakivi et al. 2021). However, it is still impossible to estimate the difference in the price of new green fuels to fossil fuels in ten years’ time.

One of the vessels between Helsinki and Tallinn is already running on LNG and the next will be in service in the summer of 2022. However, it is possible that the current fuels will not achieve sufficient emission reductions; it depends on IMO and EU regulations. If this is the case, ships can be converted to hydrogen, methanol or ammonia. Even new ships, e.g. with electricity, can be considered. However, this will require a considerable amount of capital for investments.

Perhaps the most significant change in emission reduction measures could be achieved by reducing the speed of the vessels. It is cheap and quick to implement. Therefore, in the Helsinki–Tallinn route there should be considered a speed reduction as a short-term measure. A 22-knot speed limit would reduce emissions from the passenger ferry vessels by 11% and an 18-knot speed limit by 31% (Heikkilä 2020).

Slowing down the cruising speed reduces the number of times a ship can cross the Gulf of Finland per day, thus reducing the attractivity of the service to passengers and cargo. It may even be necessary to add ships to the route to carry the same number of people and cargo, which might increase GHG emissions. When studying impact of introduction of sulphur regulation Raza et al. (2019) found that slow steaming was used only in one out of 11 shipping lines in their study. They conclude that for ro-pax and ro-ro segment bunker prices, rigorous competition and, most important, different service quality requirements have significantly restricted the potential implementation of slow steaming.

If the costs for travelling in the high-speed ro-pax ferries are raised, there is an option that the traffic divides to faster ships for the business traveller, where ticket prices are higher, and slower ships for cargo and cruise passengers. Fast transport can also be accomplished by catamarans, see e.g. Baltic Transport Journal (2021). However, their challenge is unreliability in difficult weather conditions such as storms and winter ice conditions, or even by air (helicopters and aeroplanes).

If the fuel price rises sharply, ticket prices will have to be raised significantly, which is likely to lead to ever-decreasing leisure passenger numbers. In cargo, German or Swedish lines can also compete against the line between Helsinki and Tallinn. It is also possible that some of the cargo will be containerised, and the ship type will change completely to container vessels. In that case, passengers would be transported either by slower cruise ships or fast, lightweight vessels.

In addition to the tightening environmental standards, there are other external variables, the most important of which in the coming years for Helsinki-Tallinn traffic is Rail Baltica (RailBaltica 2021). The growth of rail transport capacity in Europe, particularly the Rail Baltica ending in Estonia, will make it possible to combine sea and rail transport in a new way. Furthermore, the Covid-19 pandemic has significantly increased, e.g. direct rail connections from Asia to Europe, which will likely be part of the transport offer palette. An interesting aspect is that if the cargo arrives in containers by rail and at Rail Baltica from Poland to Tallinn by rail, container ships could be on the Helsinki-Tallinn route.

The cost impact of different measures is different. Slowing down the fleet and saving fuel will reduce costs, while new fuels, new technology and, above all, the fleet renewal will generate a lot of additional costs. After all, slowing down the fleet also reduces the available capacity per unit of time.

The most significant impact on the future of Helsinki-Tallinn traffic in next few years will be whether passenger traffic returns to the level of 2019 within a few years and continues to grow on a growth trajectory. As the traffic income between Helsinki-Tallinn is mainly dependent on passenger volumes (in total 82%), and Covid-19 pandemic did not have a noticeable effect on cargo volumes, we will concentrate on the potential recovery of the passenger volumes.

The second most significant impact on the future of the traffic will be tightening environmental regulations. The effect will be seen after 2025, but the shipping companies have to prepare themselves for that already in the next few years.

If traffic rises rapidly on a growth trajectory, new vessels and more environmentally efficient measures will be easy and likely to enter traffic. However, if the increase in traffic takes several years and vessels are hit by strict environmental restrictions, it is likely that traffic volumes will not return to previous levels, the shippers might find other routes and operating models.

These two factors, how strict the environmental regulations are and whether the volumes of passenger traffic in the Helsinki-Tallinn route return to the situation before 2020, have been chosen as a basis for the analysis. We will study the development of Helsinki-Tallinn traffic through the following framework:

1. Will passenger traffic between Helsinki and Tallinn return to pre-pandemic levels or shall the volumes stay lower?
2. How strict requirements do environmental regulations pose for future ferry traffic?

**Fig. 7:**
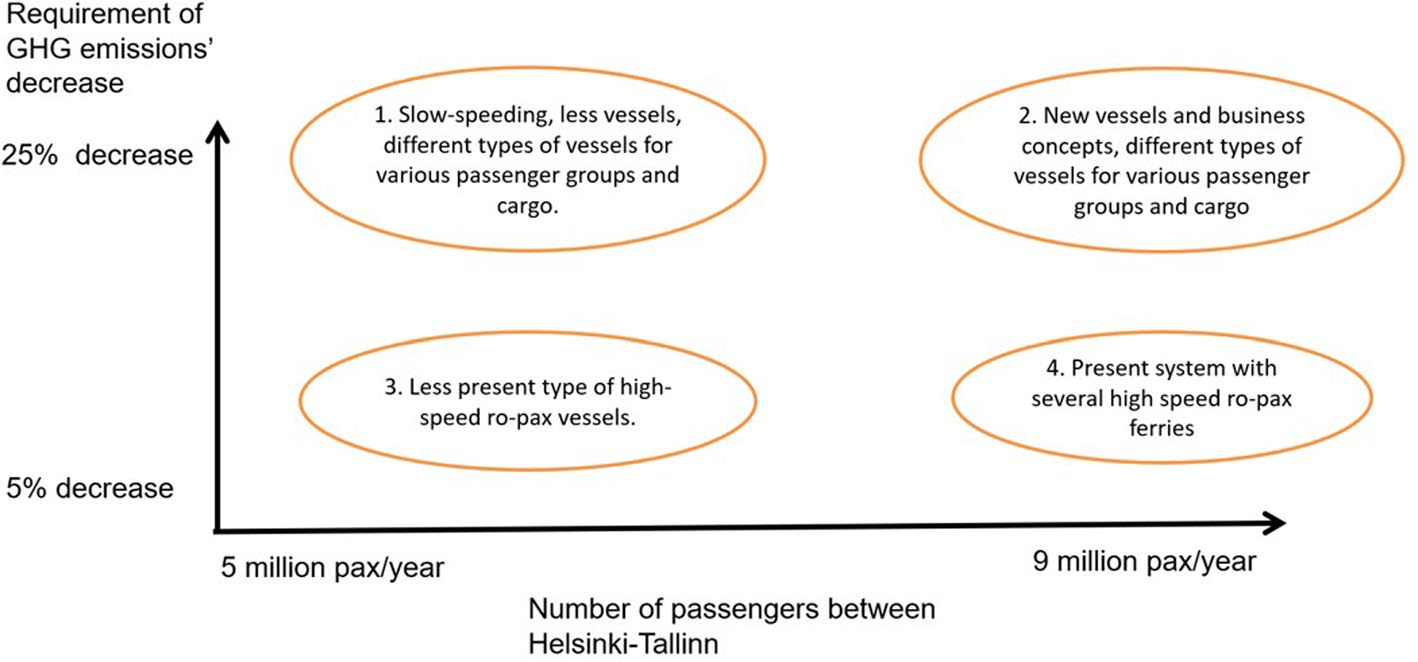
Four scenarios for Helsinki-Tallinn traffic for the year 2030. *Source*: Authors’ own compilation

Figure 7 presents the scenarios we have estimated for the situation in 2030. We chose question 1 (passenger traffic volumes) on x-axis and question 2 (effect of environmental regulations) on y-axis. It is estimated that cargo volumes will remain in the present or slightly increasing stage. The low scenario is based on the minimum level of the passenger volumes, i.e. the low number of passengers due to the start of pandemic in 2020 around 4–5 million passenger per year; and the high level is equal to the number of passengers in 2019, i.e. the peak before the pandemic broke in, around 9 million passengers per year.

The requirements of GHG emissions assumed that 5 per cent decrease can be reached with minor changes in the traffic, e.g. technological solutions to increase fuel efficiency, but 25 per cent decrease in GHG emissions can be reached only by making bigger changes in the technology, fuel or operations. These figures are assumed based on estimations of Bouman et al. (2017) of potentials of various GHG reduction measures.

The number of GHG reductions and the number of passengers are only indicative. In reality, the pressure for changing business concepts comes from emission reduction costs and income from passenger tickets and onboard sales.

### Scenario 1

If passenger traffic volumes do not increase to the level before pandemic and environmental standards become more stringent requiring more than 25% GHG decrease, the profitability of the traffic will not recover and there will likely be a major change in traffic patterns with a great fleet renewal. It is assumed that only some of the current high-speed ro-pax ferries will continue to operate due to their expensive cost structure. Instead, there may be slower cruise ships in traffic, and, above all, the growth in freight traffic will be channelled into slow ro-ro vessels. It is also possible that as freight traffic grows and Rail Baltica opens, some of the cargo will shift to containers.

### Scenario 2

If passenger traffic volumes increase to the level before pandemic and climate standards are tightened requiring more than 25% GHG decrease, these climate standards will be considered in the fleet's renewal, and there is financial capacity to renew the fleet. The new fleet is likely to use new technology and green fuels. In this case, it can be assumed that work-related passengers will still demand a fast cruising speed, but even a slower speed will suffice for recreational passengers and cargo. Therefore, it is expected that new high-speed catamaran-type vessels, with alternative fuels, will enter high-speed traffic—unless high-speed traffic is wholly shifted to air transport. The probability of this scenario is also related to the level of inflations: higher the inflation level, the more demanding it is to invest on new fleet. Therefore, in case of high inflation, the reduction on fuel consumption in new vessels has be very significant.

In addition to the fast vessels, there will also be slower cruise-type passenger and freight traffic that can reduce emissions at slower speeds.. It is assumed that there will be more vessels for also only cargo traffic that would only run between Vuosaari and Muuga, in addition to the present ro-pax traffic between the city centres. In contrast, passenger traffic will continue in the ports of Helsinki and Tallinn city centre. It is also possible that as freight traffic increases and Rail Baltica opens, some of the cargo will move to containers.

### Scenario 3

If passenger traffic volumes do not increase but remain below the 2019 level on approximately 4–5 million passengers per year for at least the next ten years, there will be extra traffic capacity in fleet, resulting in a reduction in ship capacity. It is beyond this research to consider how capacity adjustment takes place: either by one company withdrawing the traffic or number of vessels decreasing. However, it is plausible that if environmental standards do not become more stringent, on a few of the existing ro-pax ferries will continue, and growth will take place primarily on the capacity of pure cargo vessels of the Vuosaari-Muuga line.

### Scenario 4

Suppose traffic increases to or above the level of 2019 over 9 million passengers but environmental standards are not tightened for the current ro-pax vessels. In that case, it is assumed that the current traffic concept, based on mainly income from passengers and which combines passenger and cargo traffic on the same ships, will continue. In the future, it is likely that slightly larger vessels may replace present vessels. Cruise-based ships and fast passenger car ferries will continue in the city centres, while freight traffic will also increase in cargo ports, i.e. on the Vuosaari-Muuga line. In other word, there is growth in every sector.

## Conclusions

In this research, we have seen how the Covid-19 pandemic decreased the passenger volumes very much in the traffic between Helsinki and Tallinn; and caused a significant financial problem for shipping companies. As Helsinki-Tallinn traffic is particularly dependent on both passenger ticket revenue and onboard sales, the Finnish and Estonian states are supporting traffic, but it is unclear how the traffic will recover. On the other hand, the cargo traffic was practically unaffected by the pandemic.

We have also studied, how environmental regulations can have major or minor impacts on transport. It was shown that these regulations can be mitigated in several different ways, technologies, fuels or operational methods. It is also possible to switch traffic to alternative routes. Thus, how the regulations treat different types of ships, technological developments, and the price of fuels will be of great importance for the future of maritime transport.

Depending on the volumes of passenger traffic, the stringency of environmental standards, technological developments and the price of alternative fuels, we have outlined four different scenarios for future traffic. They were based on the following options: to continue the same high-speed ro-pax system as in 2019, to reduce the number of vessels, to switch to new types of ships, or to slow down the speed of the vessels or to divide traffic into faster and slower ships.

The traffic between Helsinki and Tallinn is very frequent, and the ships are very fast compared to the average in operation. Transport economics is primarily based on passengers and onboard sales, but freight traffic is also essential. In the future, it is expected that freight traffic will continue to grow, but the recovery of passenger traffic will be crucial to the future development of the traffic.

## Discussion

This study updates the previous studies of Sundberg et al*.*(2012) and Hilmola et al. (2015) by analysing the general picture of Helsinki-Tallinn traffic in the beginning of 2020s. Moreover, this study updates the future estimation of these two studies by presenting – not only one—but four possible scenarios for future development.

Moreover, this study gives more insight to the research of short-sea shipping. In “Success factors of short sea shipping” section, the literature study showed that the most critical factors of the short-sea shipping are speed and costs of the transport. In addition, it was shown that factors like cost, speed, flexibility, reliability, attractive sailing times, maintained transit times, and guarantee of delivery have big effect on the traffic.

In this research we have studied how the changing business environment, namely decreasing passenger volumes, due to Covid-19 pandemic, and increasing transport costs, due to environmental regulations, can change fundamentally the traffic concept. These factors may change volumes, costs and speed of the sea-crossing, like presented also in literature review in “Success factors of short sea shipping” section.

There are several limitations in this research. First, the new environmental regulations are still in process, and their cost effect for shipping are still unknown. In addition, the scenarios are based on literature and public statistical sources and do not view companies’ internal calculations. Finally, it is not yet known how the passenger volumes are recovering.

It is also possible that, after all, the future structure of Helsinki-Tallinn traffic will be determined by factors that are not at all dependent on emission reductions or traffic growth. These could be, for example, changes in crew costs or taxation, or possibly changes in transport modes, such as Rail Baltica.

As proposal to further research, we do not know yet, what will be the situation with these two changes, is passenger traffic returning to the previous levels and how big will the financial impact of the environmental regulations. In a few years further analysis should be made to see how the passenger traffic has recovered and how the shipping companies are answering to the tightening environmental regulation. This study will provide a basis of this further analysis, and give more insight how robust is short-sea shipping to changes in the business environment.

This study could also be compared to other short-sea shipping routes, particularly in the Southern Baltic Sea and the Mediterranean. It can be estimated that these routes will face similar challenges. Also, potential changes might be similar, even though the relative importance of passenger volumes are not so clear.

## Data Availability

All data have been required from publicly published sources. All figures are drawn by authors themselves. Data of Helsinki-Tallinn traffic is achieved from the Finnish Transport and Communications Agency Traficom by email and excel-sheets.

## Abbreviations

CCS: Carbon capture and storage
CH4: Methane
CO2: Carbon dioxide
CO2e: Carbon dioxide equivalent
ETS: Emissions Trading System
EU: European Union
GHG: Greenhouse gas
IMO: International Maritime Organization
MECP: IMO Marine Environment Protection Committee
N2O: Nitrous dioxide
Ro-pax: A roll on/roll off passenger
Ro-ro: Ro-ro is an acronym for Roll-on/roll-off
